# Supplementary material for: Chemical Composition, Fatty Acid Profile, and Optimization of the Sacha Inchi (Plukenetia volubilis L.) Seed-Roasting Process Using Response Surface Methodology: Assessment of Oxidative Stability and Antioxidant Activity
Source: Foods. 2023 Sep 12;12(18):3405. doi: 10.3390/foods12183405 (PMC10528131; doi:10.3390/foods12183405)
Supplement: Supplementary file 1 [file foods-12-03405-s001.zip › foods-2492768-supplementary.pdf]

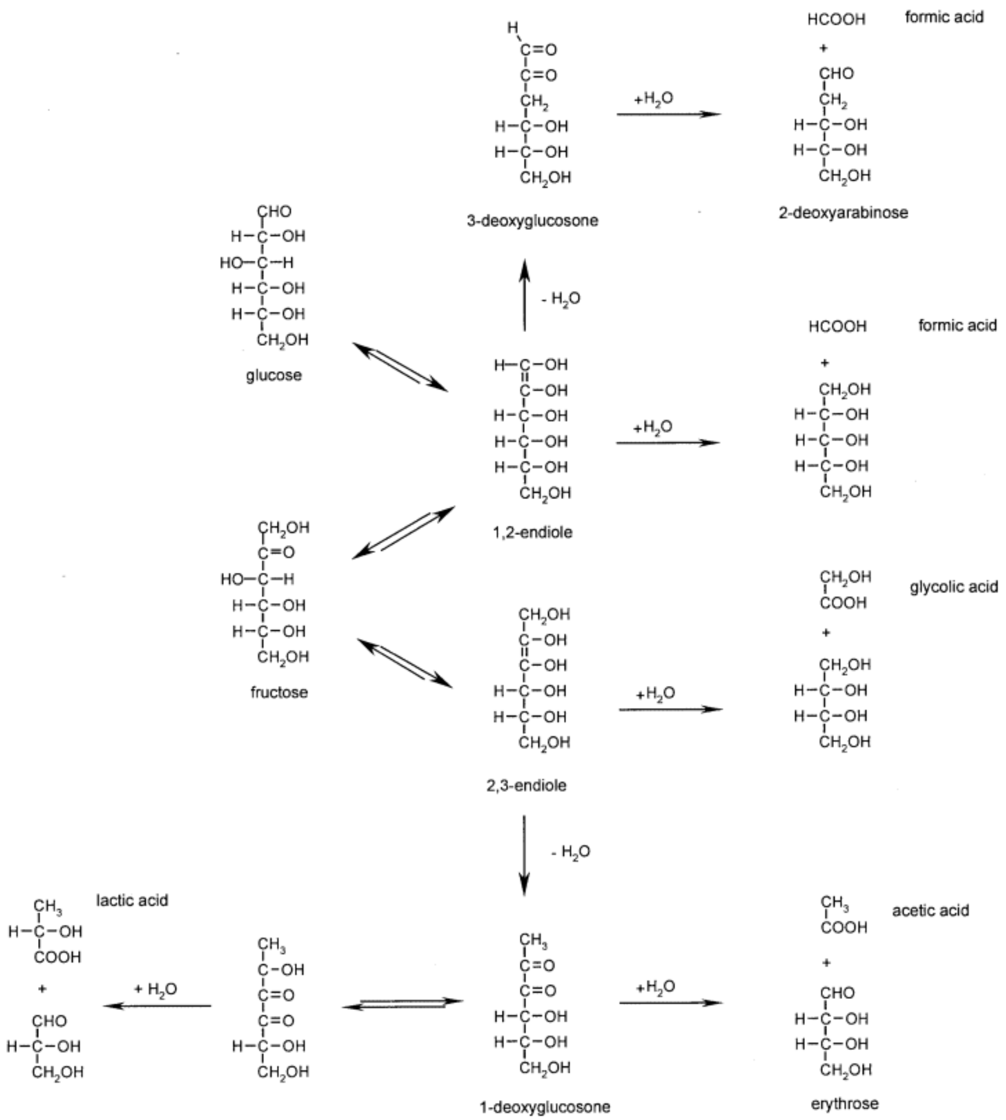


**Figura S1.** Reaction scheme for acid formation from primary therma degradation products of glucose (Ginz et al., 2000).
